# QSAR and scaffold-based optimization of HMGR inhibitors using cheminformatics and machine learning

**DOI:** 10.3389/fbinf.2026.1764859

**Published:** 2026-04-30

**Authors:** Priya Antony, Bincy Baby, Ranjit Vijayan

**Affiliations:** 1 Department of Biology, College of Science, United Arab Emirates University, Al Ain, United Arab Emirates; 2 Department of Chemistry, College of Science, United Arab Emirates University, Al Ain, United Arab Emirates; 3 Zayed Center for Health Sciences, United Arab Emirates University, Al Ain, United Arab Emirates

**Keywords:** cheminformatics, HMGR, inhibitor, machine learning, QSAR

## Abstract

Atherosclerosis, driven by elevated cholesterol levels, remains a major risk factor for cardiovascular disease. 3-hydroxy-3-methylglutaryl-coenzyme A reductase (HMGR), the rate-limiting enzyme involved in cholesterol biosynthesis, represents a validated therapeutic target. Statins are an effective class of drugs widely prescribed for HMGR inhibition; however, their prolonged use causes adverse side effects. This highlights the need for novel inhibitors with improved safety and efficacy. In this study, a comprehensive cheminformatics and machine learning approach was applied to identify and optimize potential HMGR inhibitors. A curated dataset from the ChEMBL database was analyzed through physicochemical descriptor profiling, exploratory data analysis, and principal component analysis (PCA). Murcko scaffold extraction revealed that active molecules clustered around complex cyclic frameworks enriched in aromatic and nitrogen-containing motifs. Following this, quantitative structure–activity relationship (QSAR) models were developed using various machine learning algorithms, and it was found that gradient boosting and XGBoost regressors demonstrated the best performance, with a tuned XGBoost achieving a cross-validated *R* ^2^ of 0.70. Ligand-based R group enumeration further refined promising cores, enhancing hydrogen bonding, polarity, and multiparameter optimization (MPO) scores. Four scaffolds were successfully optimized, with improved MPO values. Thus, by integrating cheminformatics and machine learning, this study provides a systematic pipeline that highlights promising scaffolds optimizing drug-likeness for the development of novel HMGR inhibitors.

## 1 Introduction

Atherosclerosis, also referred to as coronary artery disease (CAD), is a leading cause of morbidity and mortality worldwide (Björkegren and Lusis, 2022). It is characterized by elevated plasma cholesterol levels, particularly low-density lipoprotein (LDL) and triglyceride levels. Over time, these lipids accumulate within the arterial wall, triggering inflammatory responses and promoting plaque formation (Björkegren and Lusis, 2022). Progressive plaque development narrows arterial lumens and reduces vascular elasticity, ultimately leading to severe cardiovascular events such as myocardial infarction and stroke. Elevated cholesterol levels are a primary reason for CAD and cholesterol biosynthesis is tightly regulated by 3-hydroxy-3-methylglutaryl-coenzyme A reductase (HMGR), the rate-limiting enzyme of the mevalonate pathway (Friesen and Rodwell, 2004). HMGR catalyzes the reductive cleavage of HMG-CoA to mevalonate through two sequential hydride transfers, each requiring NADPH as a cofactor (Bauer, 2003). Mevalonate serves as the key precursor for isoprenoids, a diverse class of biomolecules essential for sterol biosynthesis and the regulation of cellular growth. Additional metabolites generated through the mevalonate pathway include cholesterol, heme, and farnesyl pyrophosphate (Haines et al., 2013).

In humans, HMGR is a single-chain polypeptide of 888 amino acids, with its N-terminal domain anchored to the endoplasmic reticulum membrane and its catalytic C-terminal domain localized in the cytoplasm, linked by a flexible segment (Istvan et al., 2000). Inhibition of HMGR effectively lowers plasma cholesterol concentrations, making it a validated therapeutic target. Statins, a class of potent HMGR inhibitors, competitively occupy the enzyme’s catalytic site and block mevalonate production (Istvan and Deisenhofer, 2001). This inhibition not only reduces cholesterol biosynthesis but also upregulates LDL receptors, thereby enhancing cholesterol clearance from circulation. These drugs share an HMG-like moiety, which can effectively occupy the catalytic portion of the enzyme, thus preventing HMG-CoA’s access to the active site. Thus, statins remain among the most widely prescribed medications for the treatment of hypercholesterolemia. However, despite their clinical success, prolonged statin therapy is associated with adverse effects, including hepatotoxicity, myopathy, cataracts, kidney dysfunction, increased risk of diabetes and vascular complications within the central nervous system (Ramkumar et al., 2016; Newman et al., 2019; de Pádua Borges et al., 2021). These limitations underscore the need for novel HMGR inhibitors with improved efficacy and safety profiles.

Advances in computational drug discovery provide an attractive route for identifying such compounds. Cheminformatics integrates chemical and computational methodologies to accelerate lead identification and optimization. In particular, quantitative structure–activity relationship (QSAR) modeling establishes correlations between molecular features and biological activity, enabling the prediction of novel bioactive molecules (Tropsha, 2010; Verma et al., 2010). Existing computational studies focused on a limited number of chemotypes, often without a systematic analysis of scaffold space or global optimization of drug-like properties. This often limits their ability to highlight potential scaffolds for HMGR inhibition.

In this work, we propose an integrated cheminformatics and machine learning framework for HMGR inhibitor design. The workflow involved characterizing the physicochemical property space and chemical diversity of a curated set of HMGR inhibitors; performing Murcko scaffold analysis to identify core frameworks enriched in highly active compounds; developing and evaluating machine learning–based QSAR models using Morgan fingerprints to capture non-linear structure–activity relationships;, applying ligand-based R-group enumeration, docking, and MPO-guided optimization to propose refined scaffolds with improved drug-like profiles; and finally validating the stability of selected protein–ligand complexes using 200 ns molecular dynamics (MD) simulations. Together, this integrative workflow combines data-driven modeling with structure-based design to identify and prioritise potential HMGR inhibitors for further development.

## 2 Materials and methods

### 2.1 Data collection and data cleansing

An inhibitor dataset for HMG-CoA reductase was downloaded from the ChEMBL database (ChEMBL402) (Mendez et al., 2019). Only compounds with reported IC_50_ data were used for the analysis. A data cleansing process was performed to remove duplicates and compounds without IC_50_ values. For better visualization and data processing, the IC_50_ values in the dataset were transformed into pIC_50_ values. The compounds with pIC_50_ ≥ 8 was classified as active, pIC_50_ between 6 and 8 as intermediate, and those with pIC_50_ < 6 as inactive compounds.

### 2.2 Exploratory data analysis

Eight physicochemical descriptors were computed for exploratory data analysis. These molecular descriptors include molecular weight (MW), the octanol-water partition coefficient (LogP), the number of hydrogen bond acceptors (NumHAcceptors), the number of hydrogen bond donors (NumHDonors), the number of rotatable bonds (nRot), topological polar surface area (TPSA), the number of heteroatoms (nhet), and the number of aromatic rings (nAro). In addition, principal component analysis (PCA) was applied to these physiochemical descriptors to visualize the distribution patterns and identify overlaps between the compounds (Jolliffe and Cadima, 2016).

### 2.3 Murcko scaffold extraction and analysis

To analyze the molecular structures, the RDKit package was employed to extract Murcko scaffolds from the Simplified Molecular Input Line Entry System (SMILES) representations of the compounds (Bemis and Murcko, 1996; https://www.rdkit.org/, n.d.). A custom Python function was created to convert each SMILES string into a molecular object, from which the Murcko scaffold was generated. These scaffolds were then categorized based on their average pIC_50_ values, classifying them as active, intermediate, or inactive. From the identified scaffolds, the most frequently occurring active scaffolds were selected for functional group analysis using RDKit. Specific functional groups, such as aromatic rings, amines, amides, hydroxyl groups, ethers, and others, were identified by matching the scaffold structures against predefined SMARTS patterns.

### 2.4 Molecular descriptors and QSAR model building

Morgan fingerprints were calculated to capture the chemoinformatic representation of a molecular structure. These were generated using the RDKit package and then used as features in the QSAR modeling process. To build the QSAR models, the dataset was split into a training set and a test set using an 80:20 ratio. Here, 10 machine learning algorithms were employed for developing QSAR models, including Ridge Regression, Lasso Regression, ElasticNet, Random Forest Regressor, Gradient Boosting Regressor, Extra Trees Regressor, AdaBoost Regressor, Support Vector Machine (SVR), K-Nearest Neighbors (KNN) Regressor, and XGBoost Regressor. These models were implemented using the scikit-learn library in Python. Based on the *R* ^2^ and root mean squared error (RMSE) values, XGBoost Regressor was identified as one of the top-performing models. To further optimize its performance, fine-tuning was performed using GridSearchCV. The optimized XGBoost model was subjected to 5-fold cross-validation, and the results were compared to the initial model.

### 2.5 R group enumeration using ligand designer

R-group enumeration was carried out using the Ligand Designer module in the Schrödinger Maestro suite 2024-4. Previously identified scaffolds were used as the starting point for optimization. Growth vectors were defined at solvent-exposed positions of the scaffold, and potential substitution sites were evaluated based on their orientation toward key active-site residues, including Asp690, Arg590, and Ser661 (Istvan et al., 2000; Istvan and Deisenhofer, 2001). Using the pathfinder bonds workflow, R-group substituents were systematically enumerated to explore the ligand variants. A diverse set of functional groups was introduced to enhance hydrogen-bonding interactions, adjust polarity, and modulate physicochemical properties. During enumeration, real-time multi-parameter optimization (MPO) scoring was employed to monitor the impact of substituents on drug-likeness parameters, including molecular weight, ALogP, hydrogen bond donor (HBD) and acceptor (HBA) counts, and polar surface area (PSA). Based on the literature, custom desirability profile was used with the following parameters: MW (very high importance; transitions at 300, 350, 520, 580 Da), AlogP (very high importance; transitions at 1, 2, 4, 5), PSA (very high importance; transitions at 60, 80, 130, 150 Å^2^), HBA (high importance; transitions at 4, 5, 9, 11), and HBD (high importance; transitions at 0, 1, 2, 3) (Leeson and Springthorpe, 2007; Wager et al., 2010). For each enumerated analogue, molecular docking studies and binding energy values were calculated using Schrodinger Maestro suite 2024–4.

### 2.6 Molecular dynamics simulations

To examine the dynamics and stability of the protein–scaffold complex, poses were subjected to 200 ns MD simulations using Desmond employing the OPLS4 force field (Lu et al., 2021). The complexes were placed in orthorhombic boxes and solvated with single-point charge (SPC) water molecules using the Desmond System Builder. The systems were neutralized with counterions, and a 0.15 M NaCl salt concentration was maintained. The OPLS4 forcefield was used for all calculations, and all systems were subjected to Desmond’s default eight-stage relaxation protocol before the start of the production run. For the simulations, the isotropic Martyna–Tobias–Klein barostat and the Nosé–Hoover thermostat were used to maintain the pressure at 1 atm and temperature at 300 K, respectively (Martyna et al., 1992; 1994). The short-range cut-off was set as 9.0 Å, and long-range coulombic interactions were evaluated using the smooth particle mesh Ewald method (PME) (Essmann et al., 1995). Finally, simulation trajectories were analysed to identify the stability of the protein–ligand interactions and the integrity of the complex.

## 3 Results and discussion

### 3.1 Exploratory data analysis

**FIGURE 1:**
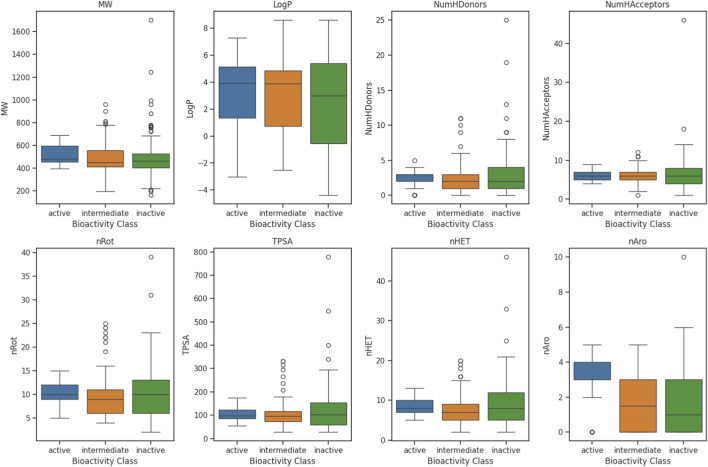
Analysis of eight physicochemical properties.

Exploratory data analysis was performed to investigate the physicochemical property ranges, distributions, and underlying patterns among bioactivity classes (active, intermediate, and inactive). Figure 1 and Table 1 summarize the analysis of eight key descriptors. All descriptors exhibit nonparametric distribution patterns, necessitating the use of the Mann-Whitney U test to assess statistical significance among bioactivity classes. Based on the results of the U-test, significant differences were observed in descriptors such as MW, NumHDonors, and pIC_50_. Active compounds generally demonstrate moderate molecular weights, balanced hydrophobicity and fewer hydrogen bond donors, suggesting a characteristic chemical space associated with bioactivity. Skewness and kurtosis analyses provided further insights into the distribution of shapes. Specifically, active compounds showed moderate skewness in MW and high positive skewness in NumHDonors, indicating that while most molecules have moderate molecular weight, some notably heavier and polar compounds are present. Additionally, high kurtosis in NumHDonors suggested a heavily tailed, leptokurtic distribution, implying that most active molecules are concentrated around fewer hydrogen donors, with occasional extreme cases.

**TABLE 1:** Summary of eight physicochemical properties and comparison between bioactivity classes.

Descriptor	Group	Max	Min	Median	Mean	Skewness	Kurtosis
MW	Active	962.30	196.24	470.51	505.26	0.76	1.91
LogP	Active	8.59	−3.03	3.94	3.18	−0.14	−0.80
NumHDonors	Active	11	0	2	2.49	2.36	9.22
NumHAcceptors	Active	12	1	6	5.93	0.86	1.78
pIC50	Active	11.69	6	8.05	8.07	0.69	1.77
nRot	Active	25	4	9	9.79	1.45	3.73
TPSA	Active	332.75	27.69	98.41	105.75	2.61	10.31
nHeteroatoms	Active	20	2	8	8.29	1.02	2.09
nAromaticRings	Active	5	0	3	2.31	−0.37	−0.95
MW	Intermediate	1701.20	162.14	452.55	468.89	2.78	11.82
LogP	Intermediate	8.08	−3.05	3.06	2.41	−0.14	−1.09
NumHDonors	Intermediate	25	0	2	3.05	3.93	19.71
NumHAcceptors	Intermediate	46	1	6	6.10	5.28	33.00
pIC50	Intermediate	6.01	5.05	5.40	5.46	0.22	−1.34
nRot	Intermediate	21	2	9.5	9.96	0.61	0.00
TPSA	Intermediate	777.98	27.69	93.94	117.93	3.94	20.37
nHeteroatoms	Intermediate	46	2	7	8.33	3.17	15.18
nAromaticRings	Intermediate	10	0	1	1.625	1.86	5.40
MW	Inactive	1243.38	264.32	483.53	523.25	1.95	4.90
LogP	Inactive	8.59	−4.39	2.77	2.59	−0.13	−1.30
NumHDonors	Inactive	19	1	3	4.13	1.87	4.03
NumHAcceptors	Inactive	18	2	6	6.41	1.27	2.58
pIC50	Inactive	5	2.82	4.65	4.40	−1.16	0.46
nRot	Inactive	39	3	10	11.23	2.02	4.88
TPSA	Inactive	548.09	37.3	116.09	143.37	1.71	3.29
nHeteroatoms	Inactive	33	2	8	9.67	1.48	2.71
nAromaticRings	Inactive	4	0	1	1.06	0.92	−0.35

### 3.2 Chemical space visualization by PCA

**FIGURE 2:**
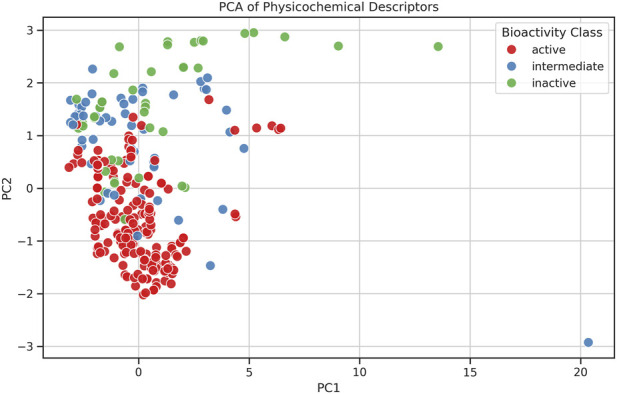
PCA visualization of physicochemical descriptors of compounds with bioactivity classification.

Based on the PCA analysis, active class compounds are found to be tightly clustered and occupy a smaller region of the chemical space, indicating less diversity in their physicochemical properties compared to intermediate and inactive compounds. Intermediate compounds occupied a broader region of the chemical space that overlapped with both the active and inactive groups, suggesting that these compounds share features with both classes. Inactive compounds were found to have a scattered distribution, suggesting the diversity and less common physicochemical characteristics of active compounds (Figure 2). Table 2 presents the eigenvalues of the eight properties, which revealed that PC1 is primarily contributed by MW, NumHDonors, NumHAcceptors, nRot, TPSA and nHeteroatoms. This represents molecular size and complexity, with high polarity and flexibility contributing significantly. PC2 mainly captures pIC_50_ and aromaticity, suggesting that biological potency strongly relates to aromatic content and polarity. PC3 primarily reflects the hydrophobicity of molecules. Chemical space visualization offers a general overview of the molecules. To conduct a detailed investigation in depth, scaffold analysis was employed.

**TABLE 2:** PCA loadings of molecular descriptors and their contribution.

Property	PC1	PC2	PC3
MW	0.38244951	−0.2248613	0.28015641
LogP	−0.1827191	−0.2762916	0.84418834
NumHDonors	0.38934695	0.18863238	−0.0023082
NumHAcceptors	0.38680817	−0.0896857	0.1307323
pIC50	−0.0499834	−0.6385372	−0.4338677
nRot	0.36804444	0.07128924	−0.0120466
TPSA	0.41991072	0.13086239	−0.0186651
nHeteroatoms	0.42237239	−0.0323548	−0.0073674
nAromaticRings	0.16265302	−0.6312614	−0.0545274
Cumulated variance (%)	59.5846798	76.127872	86.3822314

### 3.3 Murcko scaffold analysis

**FIGURE 3:**
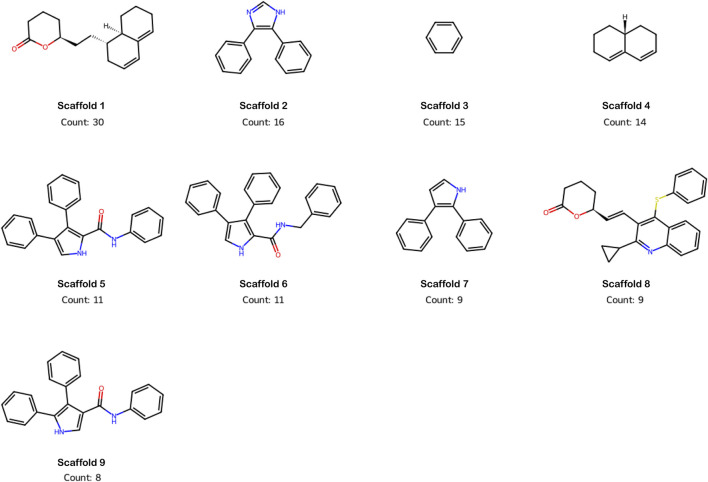
Top-ranked scaffolds in the dataset based on frequency of occurrence.

The Murcko scaffold analysis provides insights into the core structures shared among the compounds and their relationship to bioactivity. It consists of three aspects: scaffold visualization, scaffold diversity analysis, and scaffold correlation with bioactivities. Table 3 summarizes the top scaffolds identified from the dataset, which were grouped based on their bioactivity class and the corresponding pIC_50_ values. Scaffold diversity was calculated as the ratio of the number of scaffolds (Ns) to the total number of molecules (N) for each bioactivity class. The proportion of cyclic skeletons (Ncsk) was also calculated, providing insights into the molecular complexity across bioactivity classes. The Murcko scaffold diversity analysis revealed that active class molecules showed the lowest scaffold diversity (0.327), compared to the intermediate (0.571) and inactive classes (0.535). which suggests active compounds share more common core structures. Apart from this, the active class exhibits higher molecular complexity, as evidenced by the higher ratio of cyclic skeletons per scaffold (3.04), indicating that these molecules may rely on more complex, cyclic frameworks for bioactivity. Most frequently found scaffolds in the dataset are shown in Figure 3. To correlate scaffolds with bioactivity, scaffolds against bioactivity values were plotted to identify favorable scaffolds for optimization. The analysis identified several representative scaffolds, indicating their potential as promising bioactive candidates. Based on the analysis, four scaffolds (Scaffold 2, Scaffold 5, Scaffold 6, and Scaffold 7; Table 4) demonstrated high bioactivity, suggesting that these core structures have significant interactions with the target. Among the active scaffolds, scaffold 2 exhibited a notable frequency, with a count of 16. The Scaffolds 1, 4, and 9 displayed intermediate bioactivity, with scaffold 1 bearing the highest frequency in the dataset (Count: 30). These findings suggest that active scaffolds hold significant promise as candidates for drug development, while intermediate scaffolds may benefit from functional group modifications to enhance their bioactivity profile.

**TABLE 3:** Murcko scaffold diversity analysis.

Bioactivity class	Number of molecules (N)	Murcko scaffold (Ns)	Cyclic skeletons (Ncsk)	Ns/N	Ncsk/N	Ncsk/Ns
Active	211	69	210	0.327014	0.995261	3.043478
Intermediate	56	32	48	0.571429	0.857143	1.500000
Inactive	43	23	41	0.534884	0.953488	1.782609

**TABLE 4:** Murcko scaffold distribution with corresponding mean, PIC_50_, counts, and bioactivity classification.

Name	Murcko scaffold	Mean pIC_50_	Count	Bioactivity class
Scaffold1	O=C1CCC [C@@H](CC [C@H]2CC = CC3 = CCCC [C@@H]32)O1	7.4	30	Intermediate
Scaffold 2	c1ccc (-c2nc [nH]c2-c2ccccc2)cc1	8.1	16	Active
Scaffold 3	c1ccccc1	5.8	15	Inactive
Scaffold 4	C1 = CC2 = CCCC [C@@H]2CC1	7.1	14	Intermediate
Scaffolld 5	O=C(Nc1ccccc1)c1 [nH]cc (-c2ccccc2)c1-c1ccccc1	8.9	11	Active
Scaffold 6	O=C(NCc1ccccc1)c1 [nH]cc (-c2ccccc2)c1-c1ccccc1	8.9	11	Active
Scaffold 7	c1ccc (-c2cc [nH]c2-c2ccccc2)cc1	8.0	9	Active
Scaffold 8	O=C1CCC [C@@H](/C=C/c2c (C3CC3)nc3ccccc3c2Sc2ccccc2)O1	4.3	9	Inactive
Scaffold 9	O=C(Nc1ccccc1)c1c [nH]c (-c2ccccc2)c1-c1ccccc1	7.8	8	Intermediate

It was observed that all four identified active scaffolds (2, 5, 6, and 7) share structural similarity, such as aromatic rings, nitrogen-containing groups, and amide linkages, which are known to enhance binding potential through π-π stacking and hydrogen bonding. In Scaffold 2, the core structure consists of a benzene ring attached to a five-membered nitrogen-containing heterocycle and a fused benzene ring. The presence of multiple aromatic rings would enhance the binding in hydrophobic pockets through π-π stacking interactions. Scaffold 5 features a central nitrogen-containing heterocycle attached to a phenyl ring, with an amide group connected to another phenyl ring. Scaffold 6 has a nitrogen heterocycle linked to two phenyl rings and includes an amide group, with slight variations in the side chains. The amide linkages in both scaffolds will provide additional hydrogen bonding. Although Scaffolds 5 (anilide, Nc1) and 6 (benzylamide, NCc1) differ in linker type and length, both show similar potency (pIC_50_ = 8.9), indicating that, within this chemotype, small linker variations do not strongly impact activity when the core pharmacophore is maintained. Scaffold 7 has a benzene ring linked to a nitrogen-containing five-membered heterocycle and a fused benzene ring. It contains nitrogen but lacks an amide linkage. Analyzing the structure of intermediate scaffolds revealed that scaffold 1 and 4 contains polycyclic structure with fused rings without nitrogen or other functional groups, which may limit their binding interactions compared to the active compounds, whereas scaffold 9 contains an amide linkage and aromatic rings similar to active compounds but lacks additional nitrogen-containing heterocycles. The rigid polycyclic structures of these compounds are less flexible, which may explain their reduced bioactivity.

### 3.4 Morgan fingerprints and QSAR modeling

To analyze the structural characteristics of the compounds, Morgan fingerprints were generated using the RDKit package. These fingerprints capture essential structural patterns, including functional groups, that help in predicting the bioactivity. The following algorithms were used for building the QSAR model. The tested algorithms included Ridge Regression, Lasso Regression, ElasticNet, Random Forest Regressor, Gradient Boosting Regressor, Extra Trees Regressor, AdaBoost Regressor, Support Vector Regressor (SVR), K-Nearest Neighbors (KNN) Regressor, and XGBoost Regressor. Each model was trained using an 80:20 training-test split to ensure a balanced evaluation of model performance on unseen data. Performance was assessed based on the *R* ^2^ score and Root Mean Squared Error (RMSE) on the test set (Table 5).

**TABLE 5:** Comparison of machine learning models for QSAR prediction.

Model	R^2^ (train)	R^2^ (test)	RMSE (test)	Cross-validation R^2^ mean	Cross-validation R^2^ standard deviation
Ridge regression	0.90	0.44	1.17	0.52	0.07
Lasso regression	0.30	0.30	1.31	0.30	0.03
ElasticNet	0.46	0.47	1.13	0.46	0.02
Random forest	0.89	0.61	0.97	0.67	0.05
Gradient boosting	0.88	0.66	0.90	0.66	0.06
Extra trees	0.91	0.52	1.08	0.61	0.06
AdaBoost	0.78	0.64	0.93	0.64	0.05
SVR	0.76	0.53	1.07	0.44	0.08
KNN regressor	0.63	0.50	1.10	0.31	0.12
XGBoost regressor	0.88	0.64	0.93	0.66	0.06

Of the models evaluated, gradient boosting and XGBoost regressor provided the best performance. Gradient Boosting achieved an *R* ^2^ of 0.89 on the training set, 0.67 in cross-validation, and 0.67 on the test set, with a low RMSE of 0.91. Similarly, XGBoost also showed very good performance, with *R* ^2^ = 0.89 on the training set, 0.70 in cross-validation, and 0.66 on the test set, and an RMSE of 0.93. These models demonstrated a strong balance with minimal overfitting. Although both models performed well, XGBoost was selected for further optimization due to its robustness and generalization.

To improve predictive performance, hyperparameter tuning was performed on the XGBoost regressor using grid search with 5-fold cross-validation. The tuned model achieved a cross-validated *R* ^2^ of 0.70, with both training and test *R* ^2^ values of 0.66, indicating balanced performance without overfitting. Compared to the untuned model (CV *R* ^2^ = 0.67 ± 0.06), the optimized model improved accuracy and reduced variability (CV *R* ^2^ = 0.70 ± 0.05). These results confirm that hyperparameter tuning significantly enhanced both the accuracy and stability of the XGBoost model.

### 3.5 R group enumeration and and ligand design

**FIGURE 4:**
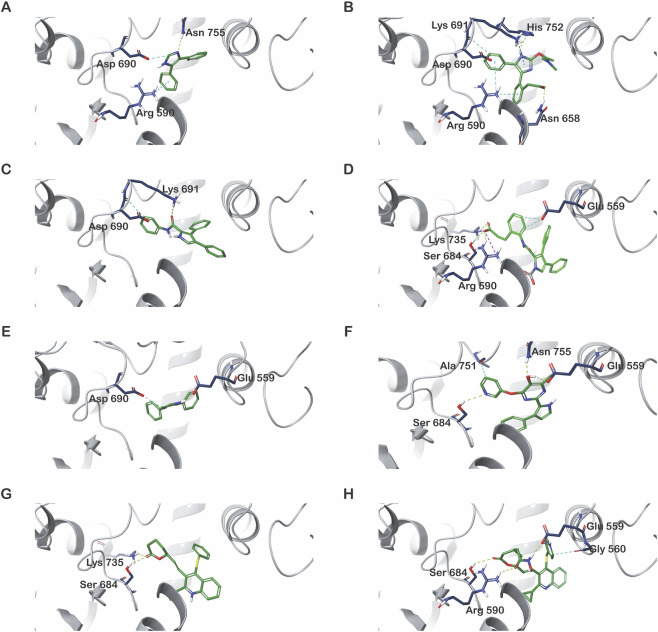
Docking analysis and interaction profile of the initial and final scaffolds. **(A)** Initial scaffold 2; **(B)** Final scaffold 2; **(C)** Initial scaffold 5; **(D)** Final scaffold 5; **(E)** Initial scaffold 6; **(F)** Final scaffold 6; **(G)** Initial scaffold 7; **(H)** Final scaffold 7.

Scaffold 2 was initially docked into the HMGR protein (1HW9) and showed weak binding with a GScore of −2.88 kcal/mol and MM-GBSA binding energy of −38.01 kcal/mol, characterized by limited interactions (Figure 4A). The initial MPO score was 0.01, with MW 220.3, PSA 28.7, and AlogP 3.7, indicating that both binding and drug-likeness were suboptimal (Table 6). To improve this profile, stepwise enumeration was performed. In the first step of enumeration, polar fragments (–OH, amide, and carbonyl groups) were added to the pyridine ring directed toward Asp690, aiming to establish hydrogen bonding. This improved hydrogen bond acceptor/donor counts but still failed to produce stable docking poses, with MPO scores remaining below 0.2. In the second round, extension toward the Asp690/Arg590 region was attempted by attaching hydroxyl and small polar fragments, which successfully introduced hydrogen bonding with Asp690 and Lys691, raising MPO to 0.37 (MW ∼294, PSA ∼86).

**TABLE 6:** Physicochemical properties and MPO scores of initial and modified HMGR scaffolds.

Scaffolds	MW(Da)	AlogP	HBD	HBA	PSA	MPO
Scaffold 5 (initial)	338.4	5.6	2	3	44.9	0.04
Scaffold 5 (final)	425.5	2.9	1	6	114.3	0.95
Scaffold 7 (initial)	402.5	5.8	0	3	40.4	0.07
Scaffold 7 (final)	476.6	4.3	2	6	102.7	0.86
Scaffold 2 (initial)	220.3	3.7	1	2	28.7	0.01
Scaffold 2 (final)	363.4	3.8	2	5	78.4	0.83
Scaffold 6 (initial)	219.3	4.3	1	0	15.8	0.01
Scaffold 6 (final)	344.4	4.3	2	5	83.9	0.76

Further optimization was performed by adding hydrophobic fragments on solvent-exposed carbons while maintaining polar contacts near Asp 690. This modification reduced steric clashes and balanced polarity. The final optimized scaffold 2 derivative showed stable interaction with Asn658, Asp690, Lys691, His752, and Arg590, while also engaging nearby hydrophobic residues. Its drug-likeness profile was significantly improved, with MW 363.4, PSA 78.4, AlogP 3.8, HBA 5, HBD 2, and MPO 0.83 (Figure 4B).

Initial docking of Scaffold 5 with HMGR showed moderate interactions with Asp690 and Lys691, but exhibited poor drug-like properties (MPO = 0.04) mainly due to high lipophilicity and insufficient polarity (Figure 4C). To address these limitations, R-group enumeration was performed on solvent-exposed vectors. In the first round of enumeration, polar substituents including amines, nitriles, and methoxy groups were introduced to probe salt-bridge formation with Asp690 and hydrogen bonding with Ser661. Even though some variants formed new interactions, these modifications often introduced steric clashes with adjacent residues and resulted in poor MPO scores. For instance, the initial analogue generated a PSA of 85.0 Å^2^ and an MPO value of 0.41. In the second design cycle, growth was directed toward the solvent-exposed region, incorporating extended polar and heteroaryl substituents. The optimized analogue was observed to interact with Glu559, while also establishing favorable polar contacts with Ser684 and Arg590 (Figure 4D). Moreover, the steric clashes were observed to be minimized. The optimized scaffold displayed improved physicochemical properties, with a molecular weight of 452.5 Da, PSA of 114.3 Å^2^, and an MPO score of 0.95, reflecting a well-balanced profile in terms of lipophilicity (AlogP = 2.9), HBD = 1, and HBA = 6 (Table 6).

In Scaffold 6, initial ligand growth was attempted towards the polar Asp690 pocket. During the first round of enumeration, small polar substituents such as hydroxyl, amide, and amino groups were introduced, but these consistently resulted in steric clashes with Asp690 and the neighboring His752 aromatic side chain (Figure 4E). As a result, docking poses failed to converge, and the MPO score remained very low (∼0.01). In the second round of enumeration, the growth strategy was re-oriented towards the solvent-exposed region. A hydroxyl-bearing substituent was introduced at the benzylic exit position, providing additional polarity without a steric penalty. This modification increased the PSA from ∼16 Å^2^ to ∼84 Å^2^, while maintaining molecular weight within the drug-like range (∼344 Da). Consequently, the MPO score improved substantially from 0.01 to 0.76. The final analogue displayed balanced hydrogen bond donors (HBD = 2) and acceptors (HBA = 5), improved drug-likeness, and favorable positioning within the active site, avoiding clashes with Asp 690 while retaining hydrophobic contacts (Figure 4F; Table 6).

**FIGURE 5:**
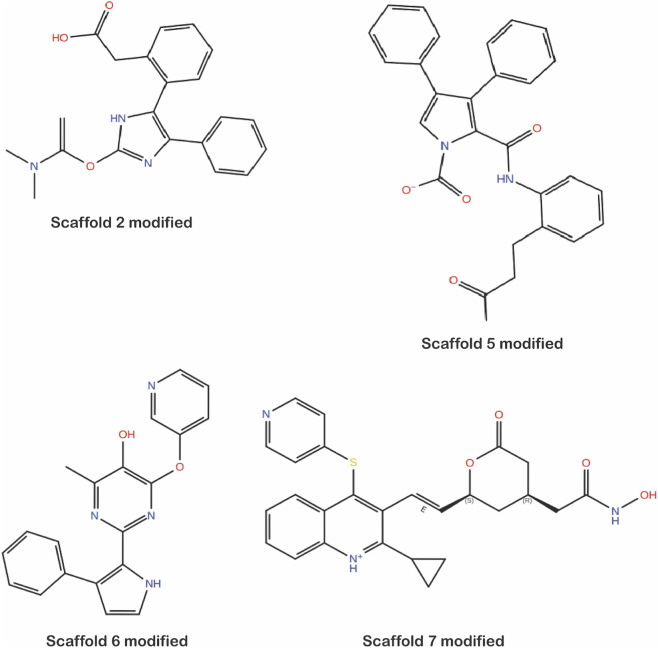
Chemical structures of modified scaffolds (2, 5, 6 and7) generated using the Ligand Designer tool.

Similarly, Scaffold 7 after initial docking exhibited promising interaction with active site residues; however, MPO score was low (0.07) due to excessive lipophilicity (AlogP 5.8), low polarity (PSA 40.4 Å^2^). To optimize drug-likeness without disturbing the pharmacophore core, substituents were systematically introduced at the solvent-exposed vector near Lys691 (Figure 4G). Addition of a hydroxymethyl group near Lys691 improved polarity and raised the MPO score to 0.67, although steric clashes remained near Glu559 and Glu560. To address the clashes, direct hydroxyl substitution was performed, which further reduced lipophilicity (AlogP 4.6) but overshot polarity (PSA 120 Å^2^), lowering MPO to 0.62. Incorporation of a direct amide cap achieved a better balance (AlogP 4.8, PSA 101 Å^2^, MPO 0.71), while a nitrile substituent was predicted to reduce PSA to ∼85–90 Å^2^ with an MPO in the 0.75–0.8 range. The most favorable variant ultimately combined improved polarity and reduced lipophilicity, yielding AlogP 4.3, PSA 102.7 Å^2^, and an MPO of 0.86, representing a significant improvement over the baseline scaffold (Figure 4H; Table 6). The improved MPO score reflects an *in silico* estimate of calculated properties rather than experimental evidence. In addition, the synthetic accessibility (SA) scores of the optimized molecules were evaluated, and the results are presented in Supplementary Table S1. The SA scores ranged from 2.67 to 4.54, indicating that the majority of the final scaffolds are predicted to be synthetically feasible. The figures of modified scaffolds are represented in Figure 5. The ligand protein interactions of initial and modified scaffolds with the protein are given as Supplementary Figures S3 and S4.

### 3.6 Molecular dynamics simulations

To understand the dynamics of the protein–scaffold complexes, MD simulations were performed for 200 ns. These simulation provide an assessment of the structural stability, flexibility and conformational behavior of the complexes. Here, root mean square deviation (RMSD), root mean square fluctuation (RMSF), and various interactions of the complexes during the simulations were assessed. RMSD of the Cα atoms of the complexes determines the average change in the displacement of the Cα atoms of the protein relative to the reference frame. It was observed that in all four complexes, after an initial rise, complexes converged and reached a stable RMSD below 3 Å (Supplementary Figure S1A-D). The complex involving Scaffold 5 reached a stable state after 50 ns, whereas the Scaffold 7 complex exhibited some degree of deviation during the simulation. Nonetheless, all three complexes maintained stable equilibrated structures throughout the simulation period with RMSD below 3 Å (Supplementary Figure S1 B and D).

**FIGURE 6:**
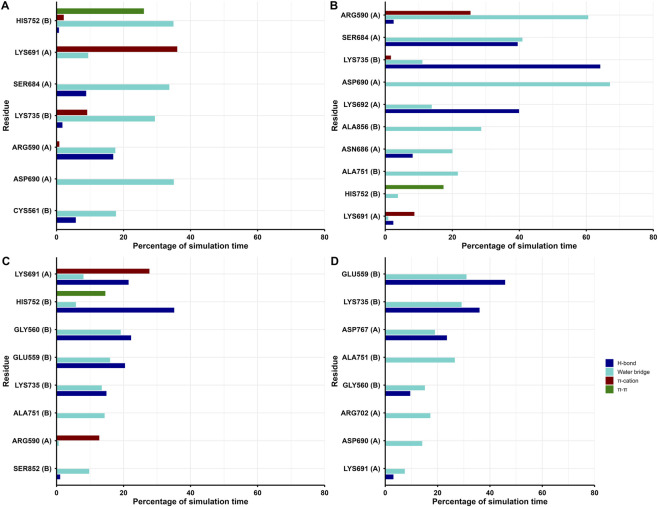
Polar residues of HMGR that interact with the scaffolds over the course of the 200 ns simulations: **(A)** Scaffold 2, **(B)** Scaffold 5, **(C)** Scaffold 6, **(D)** Scaffold 7. Different interactions are indicated by the colors (Hydrogen bonds -blue; π–cation -brown; π–π -green, and water bridge-blue).

**FIGURE 7:**
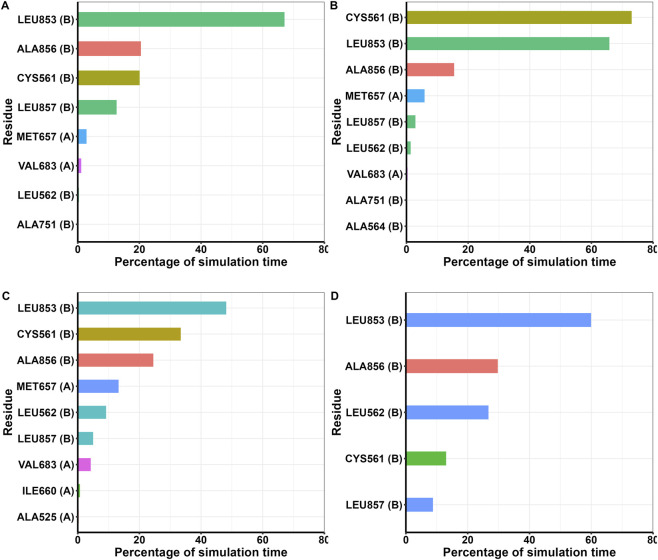
Hydrophobic residues of HMGR that interact with the scaffolds over the course of the 200 ns simulations **(A)** Scaffold 2; **(B)** Scaffold 5, **(C)** Scaffold 6, **(D)** Scaffold 7.

To determine the flexibility of the protein during the simulation period, the RMSF of Cα atoms were calculated and plotted. It indicates the residue level fluctuations within the protein during the simulation. As expected, most of the fluctuations were observed in the loops and linker regions, while well-structured α-helices and β-sheets exhibited low RMSF profiles (Supplementary Figure S2A-D). Stable binding of the scaffolds in the active site of HMGR protein was determined by the interactions, including hydrogen bonds, hydrophobic bonds, ionic bonds and water bridges. During the course of the 200 ns simulations, it was observed that numerous interactions were intermittently formed and broken between the complexes. The main interactions arose from hydrogen bonds and water-mediated bridges with occasional π–cation and π–π contacts. In all systems, key catalytic and binding-site residues such as Glu559, Gly560, Arg590, Ser684, Asp690, Lys691, Arg702, Lys735 and Asp767 show high interaction times, indicating that they remain engaged with the ligands for a large fraction of the 200 ns trajectory. These residues are involved in the active site region and the catalytic motif of the HMGR protein (Figures 6A–D). In addition to polar contacts, the MD trajectories showed persistent hydrophobic interactions between all four scaffolds and residues lining the lipophilic pocket of HMGR (Figures 7A–D). In particular, Cys561, Leu562, Met657, Val683, Leu853, Ala856 and Leu857 display high interaction times, indicating that the non-polar cores of Scaffolds 2, 5, 6 and 7 remain deeply buried in the active-site cavity. Although these compounds are scaffold design hypotheses, MD simulations and interaction networks demonstrate that all four scaffolds remain stably bound within the HMGR active site. These findings support the stability and consistency of the predicted binding modes.

## 4 Conclusion

This study combined cheminformatics and machine learning to identify and refine potential inhibitors of human HMGR. By integrating chemical space exploration, scaffold analysis, and QSAR modeling, we identified structural skeletons closely associated with bioactivity and drug-likeness. Even though this study is limited by the size of the data, the chemical diversity of the dataset, and the lack of experimental validation, the tuned XGBoost model shows consistent performance during the analysis. Therefore, the QSAR model should be regarded as an early-stage, hypothesis-generating tool for prioritizing scaffolds. Scaffold mining revealed that active molecules tend to share more complex cyclic frameworks, suggesting preferred chemotypes for inhibition. Using structure-guided R-group enumeration, key scaffolds were systematically optimized to enhance polarity and reduce lipophilicity while preserving crucial binding interactions within the enzyme’s catalytic site. However, this study is limited by the size of the data, the chemical diversity of the dataset, and the lack of experimental validation. Nonetheless, this integrative approach demonstrates how QSAR modeling and structure-based design can converge to guide the discovery of novel HMGR inhibitors with improved therapeutic potential. Future studies, including *in vitro* and *in vivo* analysis, could validate the findings of this study.

## Data Availability

The original contributions presented in the study are included in the article/Supplementary Material, further inquiries can be directed to the corresponding author.
