# Supplementary material for: QSAR and scaffold-based optimization of HMGR inhibitors using cheminformatics and machine learning
Source: Front Bioinform. 2026 Apr 30;6:1764859. doi: 10.3389/fbinf.2026.1764859 (PMC13171508; doi:10.3389/fbinf.2026.1764859)
Supplement: Supplementary file 1 [file DataSheet1.pdf]

QSAR and scaffold-based optimization of HMGR inhibitors using cheminformatics and machine learning

Priya Antony[1], Bincy Baby[2], Ranjit Vijayan[1,3,*]

[1] Department of Biology, College of Science, United Arab Emirates University, PO Box 15551 Al Ain, United Arab Emirates

[2] Department of Chemistry, College of Science, United Arab Emirates University, PO Box 15551 Al Ain, United Arab Emirates

[3] Zayed Center for Health Sciences, United Arab Emirates University, PO Box 15551, Al Ain, United Arab Emirates

*** Correspondence:**
Corresponding Author
ranjit.v@uaeu.ac.ae

Supplementary Material

Supplementary Figures

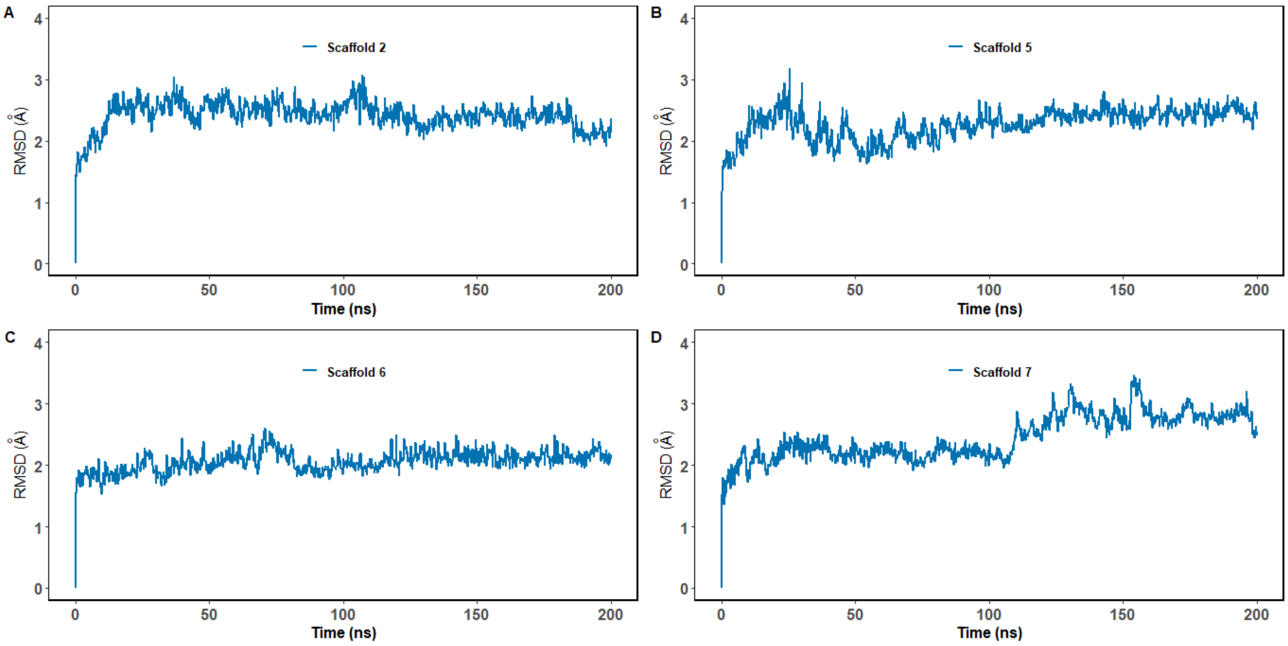

Supplementary Figure 1. The root mean square deviation (RMSD) of Cα atoms of HMGR in 200 ns. MD simulations of the HMGR complexed with (A) Scaffold 2, (B) Scaffold 5, (C) Scaffold 6, and (D) Scaffold 7.

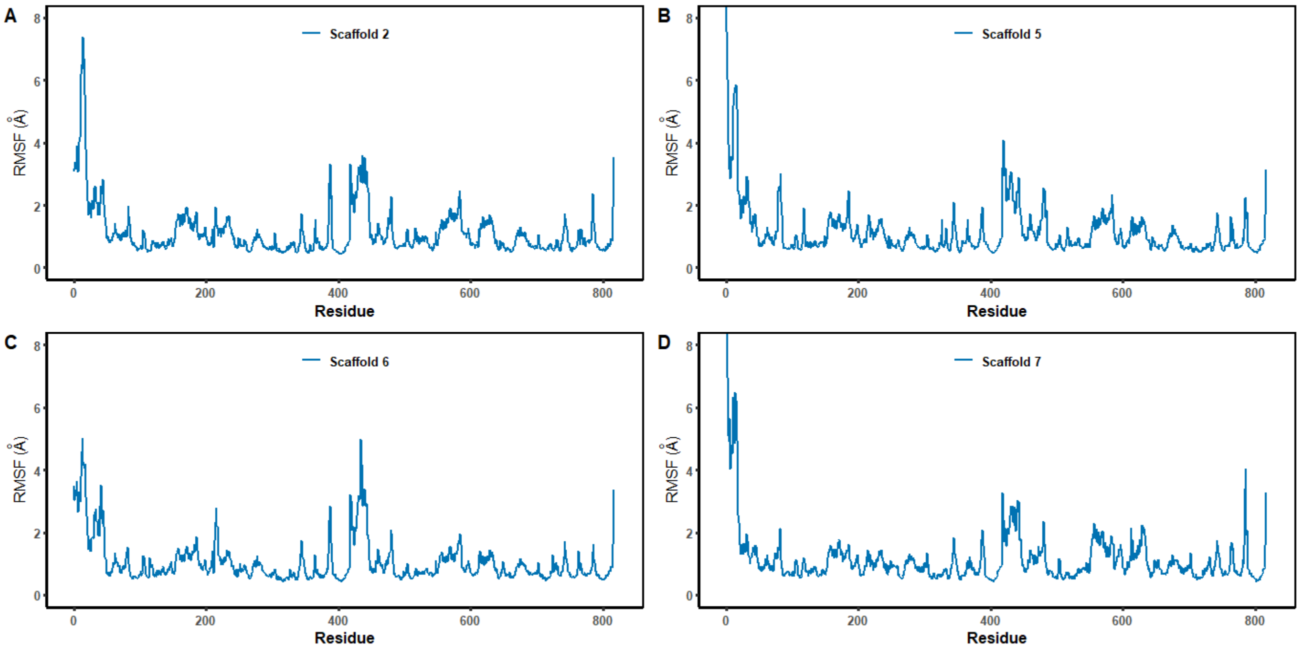

Supplementary Figure 2. The root mean square fluctuations (RMSF) of Cα atoms of HMGR in 200 ns. MD simulations of the HMGR complexed with (A) Scaffold 2, (B) Scaffold 5, (C) Scaffold 6, and (D) Scaffold 7.

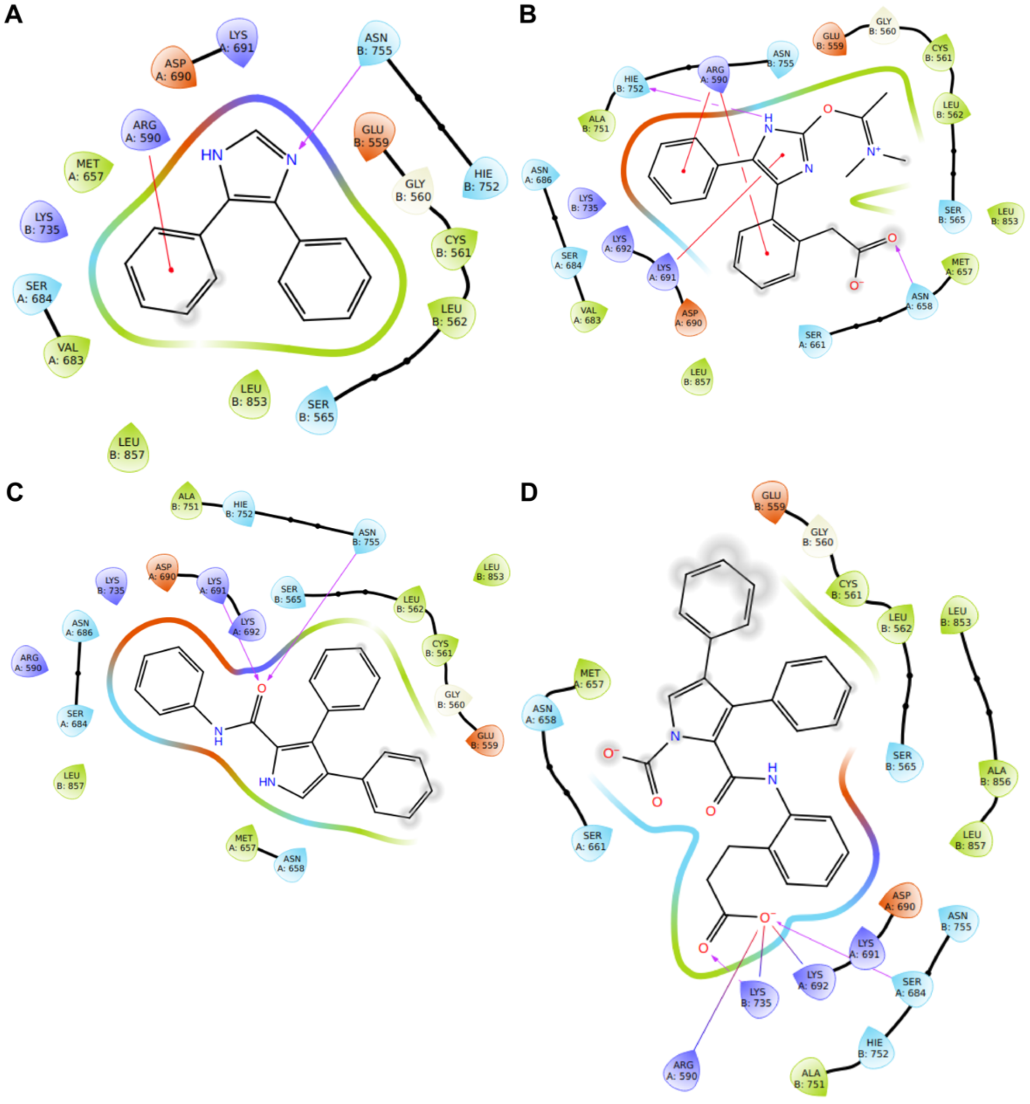

Supplementary Figure 3. Ligand–protein interaction diagrams of the initial and optimized scaffolds. (A) Initial Scaffold 2, (B) Final Scaffold 2, (C) Initial Scaffold 5, (D) Final Scaffold 5.

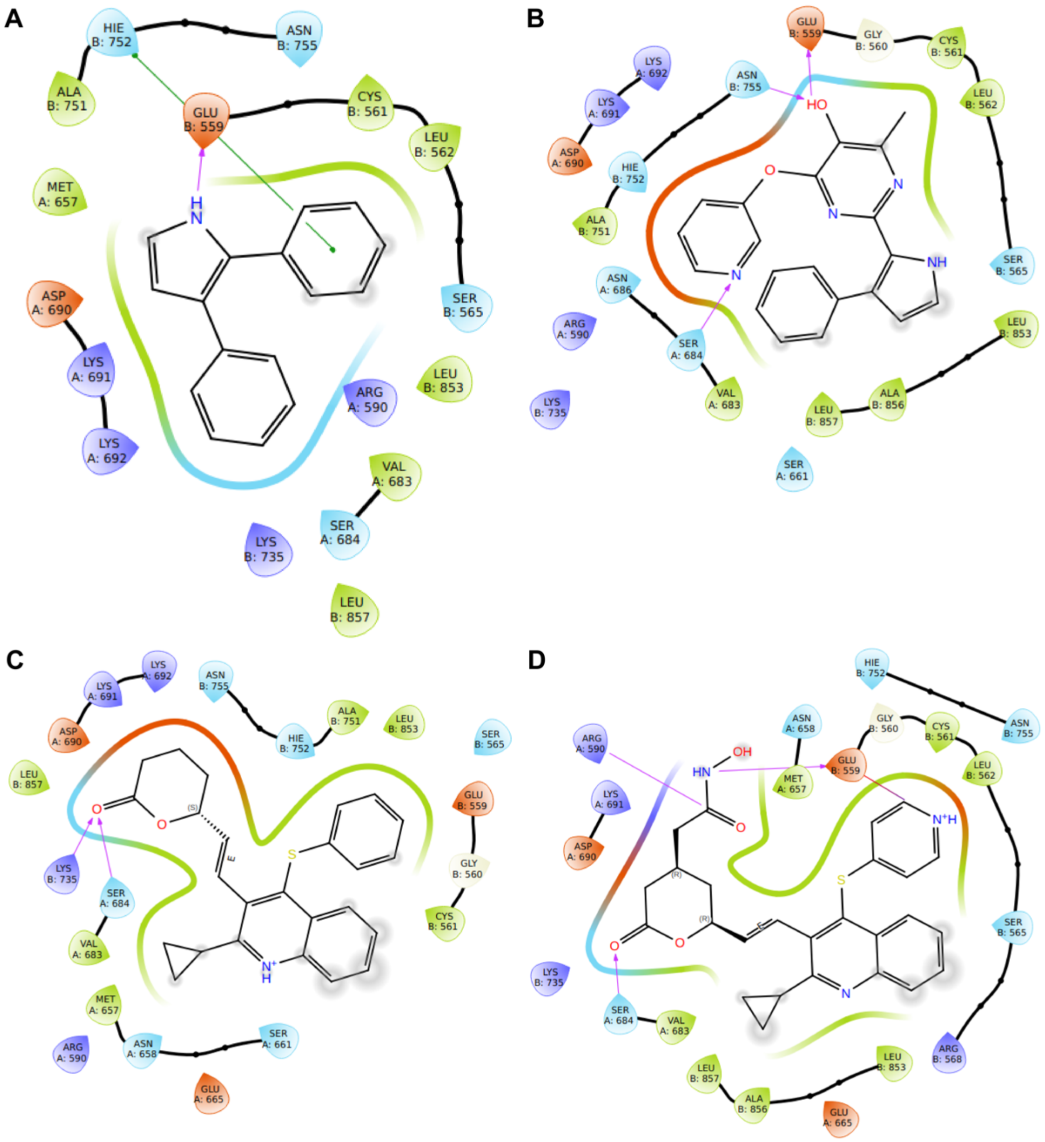

Supplementary Figure 4. Ligand–protein interaction diagrams of the initial and optimized scaffolds. (A) Initial Scaffold 6, (B) Final Scaffold 6, (C) Initial Scaffold 7, (D) Final Scaffold 7.

Supplementary Table

Supplementary Table1: Synthetic accessibility score (S_A Scores) of modified scaffolds.

Sl.No:	Scaffold ID	SA_Score
1	Scaffold 2	3.26
2	Scaffold 5	2.88
3	Scaffold 6	2.67
4	Scaffold 7	4.54
